# The Potential Role of Quorum Sensing in Clonal Growth and Subsequent Expansion of Bone Marrow Stromal Cell Strains in Culture

**DOI:** 10.1155/2019/1579102

**Published:** 2019-07-31

**Authors:** Maurizio Alimandi, Luca Pierelli, Valentina Pino, Stefano Gentileschi, Benedetto Sacchetti

**Affiliations:** ^1^Department of Clinical and Molecular Medicine, Sapienza University of Rome, 00161 Rome, Italy; ^2^Department of Experimental Medicine, Sapienza University of Rome, 00161 Rome, Italy; ^3^Università Cattolica del Sacro Cuore, Istituto di Clinica Chirurgica, 00168 Rome, Italy; ^4^Fondazione Policlinico Universitario “A. Gemelli” IRCCS, Dipartimento Scienze della Salute della Donna e del Bambino, Unità di Chirurgia Plastica, 00168 Rome, Italy; ^5^Department of Molecular Medicine, Sapienza University of Rome, 00161 Rome, Italy; ^6^Department of Science, University Roma Tre, Rome 00146, Italy

## Abstract

Clonal development (clonogenicity) is an inherent property of a subset of postnatal bone marrow (BM) adherent stromal mesenchymal stem cells (MSCs) from which a multipotent progeny develops in culture. Our data suggest that clonogenicity and BM-MSC expansion are two distinct biological events. This hypothesis is based on the following observations: (1) the beginning of clonal growth is a property strictly dependent on serum and independent of the social context, (2) the expansion of individual clone is influenced by events deriving from a social context during initial growth, (3) clonogenic cells grown in a social context in presence of serum can emancipate themselves to generate a secondary different progeny, and (4) the ability of socially generated clones to develop an inherent potential for further growth suggests that quorum sensing may operate in BM-MSC cultures and determine the potential growth of clonal strains.

## 1. Introduction

In biology, quorum sensing (QS) is defined as a process by which cells are able to detect the accumulation of released signals and change their behavior when signal concentration exceeds threshold levels. QS in prokaryotes is based on the “communication between bacterial cells” necessary to organize and regulate internal biological processes within a cell, but that depends on population density [1]. This mechanism allows in many bacteria species to coordinate behaviors in the entire population [2–4]. This interbacterial communication system utilizes small diffusible molecules (inducers) to regulate bacterial gene expression in accordance to population density. QS peptides are an important class of bacterial peptides having multiple effects on human host cells. Colonization of the large intestine in humans and cattle by enterohemorrhagic *Escherichia coli* involves cross-communication of biochemical signaling systems between bacterial pathogens and eukaryotic host cells [5–9].

Recently, it has been discovered that this mechanism is also able to influence mammalian host cells by promoting tumor cell invasion and angiogenesis *in vitro*, implying that QS are working *via* epigenetic mechanisms to stimulate cancer stem cell migration and normal stem cell differentiation for angiogenesis of vascular endothelial cells [10–13]. Previous studies demonstrated the existence of sophisticated mammalian cell-to-cell communications pathways regulating QS mechanisms for immune responses [14], including self/nonself T cell discrimination [15]. This circuit of intercellular communication in mammalian cells coordinates multicellular gene expression [16, 17], and cytokines are good candidates as inducers of QS effects such as migration, proliferation, and differentiation [14, 18]. In this scenario, and in analogy with bacteria, we wanted to verify the existence of any QS behavior in initial clonal growth and subsequent expansion of mammalian postnatal marrow adherent stromal mesenchymal stem cells (MSCs) in culture.

Bone marrow (BM) stromal stem cells are cells residing in the medullary cavity of the bone [19]. Also known as BM skeletal stem cells (BM-SSCs) or BM mesenchymal stem cells (BM-MSCs), they are specifically defined and identified only by *in vivo* transplantation assays [20, 21]. BM-MSCs are otherwise noted for the ability to initiate clonal growth (colony formation) and to generate an expanding, multipotent progeny in culture [20–22]. Thus, marrow stromal cell population contains precursor cells capable of proliferation, renewal, and differentiation into several phenotypes [19]. Even after extended culture, marrow stromal mesenchymal cells can form bone, fibrous tissue, adipose tissue, and hematopoiesis-supporting reticular stroma [19, 23–28] when implanted in conventional *in vivo* differentiation assays. It is also known that Colony-Forming Unit Fibroblasts (CFU-Fs) derived from BM have osteogenic potential. Indeed, populations of marrow stromal skeletal CFU-Fs can differentiate into functional osteoblasts and form the bone *in vivo* [20, 21]. Strategies for cell therapy using MSCs are often based on common, unverified assumptions, without considering their specific biological properties, their commitment, or their *in vitro* biological behavior. This belief betrays and reflects our poor understanding of inherent cell properties, roles, and identity, ignoring all the environmental factors influencing or sometime determining MSC behavior, during their *in vitro* growth, and subsequently once transplanted *in vivo*.

## 2. Materials and Methods

### 2.1. Preparation of BM Single Cell Suspension and Culture

BM-MSCs were obtained from iliac crest marrow aspirates of adult healthy donors (age range 20-60 years). Human subjects provided us with oral assurance of their willingness to be enrolled in the research study. The overall study on human tissues was approved by the Research Ethics Committee of Istituto Superiore di Sanità of Rome (approval date September 20, 2016; Prot. PRE-686/16). BM aspirates (0.5-1 cm^3^) were mixed with 5 ml of ice-cold *α*-modified minimum essential medium (*α*-MEM, Life Technologies, NY, U.S.A.) containing 100 U/ml sodium heparin (Fisher Scientific, NJ, U.S.A.). Cells were centrifuged at 135g for 10 minutes, and pellets were resuspended in fresh *α*-MEM. Bone marrow cell preparations from aspirate were consecutively passed through 16 and 20 gauge needles to disrupt cell aggregates. Cell suspensions were filtered through a 40-nylon cell strainer (Becton Dickinson, San Diego, CA, USA) to remove any remaining cell aggregates. Cells were plated in standard condition basal medium *α*-MEM added with 20% Fetal Bovine Serum (FBS, Invitrogen, Carlsbad, CA, USA), 2 mM L-glutamine (Invitrogen, Carlsbad, CA, USA), 100 U/ml penicillin (Invitrogen, Carlsbad, CA, USA), and 100 *μ*g/ml streptomycin (Invitrogen, Carlsbad, CA, USA) or with a serum-deprived medium (SDM) supplemented with 10 ng/ml Platelet-Derived Growth Factor (PDGF-BB, R&D systems, Minneapolis, MN, USA), 10 ng/ml Epidermal Growth Factor (EGF, R&D systems, Minneapolis, MN, USA), and fibronectin-coated substrates (Sigma-Aldrich, St. Louis, MO, USA).

### 2.2. Colony-Forming Efficiency (CFE) Assay

For multiclonal cultures, formed under “social conditions,” nucleated cells from fresh marrow aspirates were seeded into 100 mm dishes (Becton Dickinson Biosciences, San Diego, CA, USA) at a clonal density of 8.3 × 10^3^ nucleated cells/cm^2^ [21] to assay for primary CFU-Fs. To assay for secondary CFU-Fs, adherent primary MSCs from BM-derived clonal cultures were seeded into 100 mm dishes at a clonal density of 1.6 cells/cm^2^. For single colonies formed under “isolation conditions,” cells were seeded in 96-well plates at limiting density. Formation of discrete colonies was scored after 14 days. After 14 days, the CFE was determined by counting the number of Giemsa-stained colonies: number of colonies formed [(>50 cells)/number of cells initially inoculated] × 100. Assays were done at least in triplicate. Individual colonies (clones) were isolated and expanded from multiclonal primary or secondary CFU-F cultures using cloning cylinders (Sigma-Aldrich, St. Louis, MO, USA). Alternately, multiclonal cultures were passaged on day 14.

### 2.3. Microscopy and Imaging of Cells in Culture

Samples of clonal nucleated cells from unsorted human BM were plated and cultured in 100 mm dishes (8.3 × 10^3^ cell/cm^2^). The lipophilic fluorescent carbocyanine cell tracking dye DiI-Ac-LDL (Invitrogen, Carlsbad, CA, USA) was used as per the manufacturer's recommendations. Confocal fluorescence images-stacks of developmental stages of CFU-F from human BM stromal-labelled clonal cell to colony in culture were obtained using the Leica TCS SP5 confocal laser scanning microscopy system (Leica Microsystems, Mannheim, Germany) using the HeNe 543 nm and the Ar 488 nm laser lines for visualizing the red (DiI) or green (GFP) vital fluorochromes, respectively.

Time lapse brightfield light microscopy images of the development of an individual colony in human BM aspirate culture were obtained using a Zeiss Axiophot microscope (Carl Zeiss, Germany). Phase contrast images were acquired every 1-3 days.

### 2.4. Analysis of Cell Proliferation

For proliferation kinetics of MSCs, the cumulative population doubling (PD) levels were calculated from the cell count by the use of the following equation: PD = Log (*N*_*t*_/*N*_0_) × 3.33, where *N*_0_ cells are the number initially seeded and *N*_*t*_ cells are the number harvested. The living cell count was performed in triplicate in a hemocytometer by means of trypan blue dye exclusion (Sigma-Aldrich, St. Louis, MO, USA).

### 2.5. Flow Cytometry

For fluorescence-activated cell sorting (FACS), cells from primary BM MSC-derived clone cultures were pelleted, resuspended in Ca^2+^Mg^2+^-free PBS and expression of markers was assessed using a FACSCalibur flow cytometer (Becton Dickinson Biosciences, San Diego, CA, USA). Data were analyzed with the use of Cell-Quest Pro software (version 6.1, Becton Dickinson Biosciences, San Diego, CA, USA).

### 2.6. Reagents

Antibodies for flow cytometry are listed in Table 1.

### 2.7. Lentiviral Vectors

In some experiments, BM-MSCs from multiclonal cultures were transduced with green fluorescent protein (GFP)-lentiviral vectors. Lentiviral vectors for GFP expression were produced and used as described [29].

### 2.8. Statistical Analysis

Statistical analysis was performed by one-way ANOVA and subsequently by Bonferroni posttests. Results are expressed as mean ± standard deviation (SD). Differences are considered statistically significant at ^∗^*p* ≤ 0.05.

## 3. Results

### 3.1. Developmental Phases of an MSC Colony from a Single BM CFU-F

**Figure 1:**
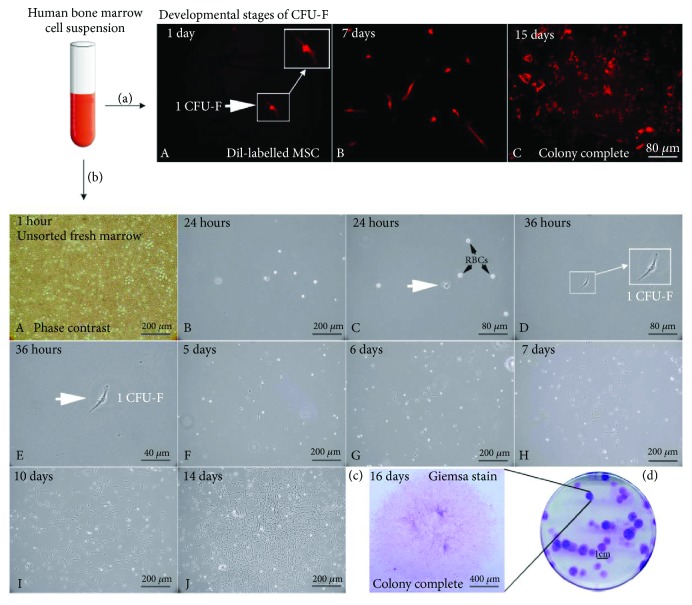
Growth phases from a BM CFU-F to a BM-MSC colony. (a) Developmental stages of primary CFU-F from human BM stromal DiI-labelled clonal cell to colony in culture (A–C) as observed by fluorescence microscopy (*n* = 5). (b) Development of an individual colony in human BM aspirate culture as observed by light microscopy (*n* = 5). Time lapse microscopy of isolated clonal cells (white arrow) from BM after explant: cell division and migration (days 1-7); define territory and size of the forming colony; and subsequent proliferation abilities within the colony [days 7-15; (A–J)]. Establishment of complete colonies as demonstrated by Giemsa staining (c, d). Results are derived from 5 independent experiments and from 5 different donors. DiI: 1,1′-dioctadecyl-3,3,3′,3′-tetramethylindocarbocyanine perchlorate; CFU-F: Colony-Forming Unit Fibroblast; RBCs: Red Blood Cells. All data shown are representative results derived from one of at least three independent experiments. Scale bars represent 40 *μ*m, 80 *μ*m, 200 *μ*m, and 400 *μ*m and 1cm as indicated.

To investigate the development of early colonies, single DiI-labelled stromal BM-MSCs [days 1-15; Figure 1(a), A–C] from fresh unsorted marrow aspirates were seeded and cultured into 100 mm dishes at 8.3 × 10^3^ clonal density (total nucleated marrow cells/cm^2^) in standard condition and monitored by time-lapse microscopy. This analysis documented the existence of two distinct developmental phases of BM-MSC characterized *in vitro*. In the first phase, cell division and migration [days 1-7; Figure 1(b), A–H] defined boundaries and size of the forming colony. The second phase was characterized by stationary proliferation [days 7-15; Figure 1(b), H–J] leading first to increased cell density within colonies, then by filling-in space, to establish a territorially discrete, but internally continuous, monolayer [day 16; Figure 1(c)], to finally form a colony as we know it in culture dishes [Figure 1(d)].

### 3.2. Initial Clonal Growth Depends on Serum

**Figure 2:**
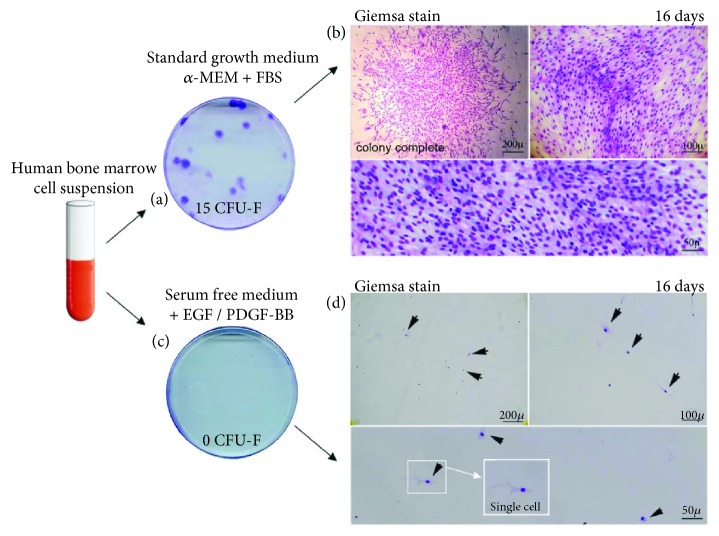
Clonal growth depends on serum. Samples of clonal nucleated cells from unsorted human BM were plated and cultured in 100 mm dishes (8.3 × 10^3^ cell/cm^2^, *n* = 5). Photographs of culture dishes showing proliferation of primary BM CFU-F, cultured in standard CFU-F growth medium supplemented with 20% FBS (a) or cultured in SDM and supplemented with EGF/PDGF (10 ng/ml) (c). After 2 weeks of culture, well-developed colonies were observed in serum-supplemented cultures (a, b). In contrast, no colony developed in SDM; single cells remained attached with an intact nucleus and an intact cytoplasm (arrows), for at least 2 weeks, as demonstrated by Giemsa staining, but did not proliferate or generate any clone in serum-free medium (c, d). Results are derived from 5 independent experiments and from 5 different donors. FBS: Fetal Bovine Serum; EGF: Epidermal Growth Factor; PDGF-BB: Platelet-Derived Growth Factor. Scale bars represent 50 *μ*m, 100 *μ*m, and 200 *μ*m.

To investigate clonal BM-MSC development, we prepared primary cultures from fresh unsorted marrow, seeding nucleated cells at a clonal density of 8.3 × 10^3^ cells/cm^2^ in a 100 mm Petri dish, either in standard conditions or in SDM. SDM is a medium able to ensure stromal strain proliferation at nonclonal densities, which can be obtained by seeding >10^5^ total nucleated cells/cm^2^ from fresh marrow aspirates. Discrete colonies of >50 cells developed only in the presence of serum [Figures 2(a) and 2(b)]. In serum-free medium, cultured cells remained viable for 14 days but failed to generate colonies [Figures 2(c) and 2(d)].

### 3.3. Effect of Social Conditions on CFU-F Growth

**Figure 3:**
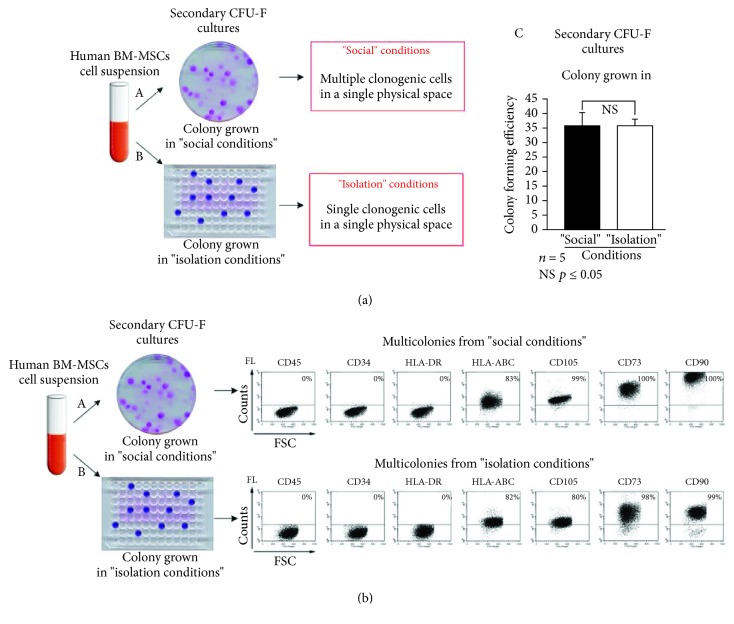
Social conditions on CFU-F growth. (a) To assay for secondary CFU-Fs, samples of nucleated cells from primary BM-MSC cultures were plated and cultured either in 100 mm dishes (density at 1.6 cells/cm^2^, where cells are physically isolated in a common environment: “social conditions”; A) or in 96-well plates at cloning dilution (where single cells are in single wells: physical and biochemical “isolation”; B) in FBS-supplemented culture medium. No differences (C) were observed between numbers of BM-MSC colonies either in “isolation” (number of CFU-Fs) or under “social” conditions (number of CFU-Fs). (b) Flow cytometric analysis of a multiclonal strain obtained by combining multiple primary colonies. No significant (NS) differences were observed between BM-MSCs obtained by combining multiple primary colonies from “social conditions” (A) or “isolation conditions” (B). Note the high/bright expression of multiple markers of BM-derived CFU-Fs (and “mesenchymal stem cells”), CD73, CD90, and CD105 and low expression of HLA-ABC. Endothelial (CD34) and hematopoietic markers (CD45, HLA-DR) are negative. Results are expressed as the mean ± SD (*n* = 5). ^∗^*p* ≤ 0.05. Results are derived from 5 independent experiments and from 3 different donors. FL: Fluorochrome; FSC: Forward Scatter.

To investigate BM-MSC clonogenic efficiencies, we prepared secondary cultures from primary BM-MSC cultures. We plated 100 secondary BM-MSCs at 1.6 cells/cm^2^ in a 100 mm Petri dish or at a limiting density in 96-well plates in standard conditions. Identical CFE [Figure 3(a), A–C] was observed when multiple clonogenic cells were plated in a single physical space compartment in a 100 mm Petri dish (“social conditions”) or when single clonogenic cells were seeded in a single physical space compartment at a limiting density in 96-well plates (^≤^1 cell in 1 space compartment; “isolation conditions”). This indicated that the capacity to initiate clonal growth is a context-independent property of a defined subset of BM-MSCs. Although no difference was observed in the number of colonies in the two types of culture, the total number of cells generated in social context cultures was more than 2-fold higher compared to that in cultures of cells generated at clonal density (data not shown). BM-MSC multicolonies from “social conditions” or “isolation conditions” exhibited a typical MSC phenotype [Figure 3(b), A and B], as defined by the International Society for Cellular Therapy (ISCT) [22], being strongly positive for CD73, CD90, and CD105 and negative for the hematopoietic markers CD34 and CD45. The flow cytometry analysis of immune-related markers confirmed the constitutive expression of HLA-A, HLA-B, and HLA-C antigens and the absence of HLA-DR [Figure 3(b), A and B].

### 3.4. Clonal Expansion Is a Biological Property of MSC Acquired in a Social Context

**Figure 4:**
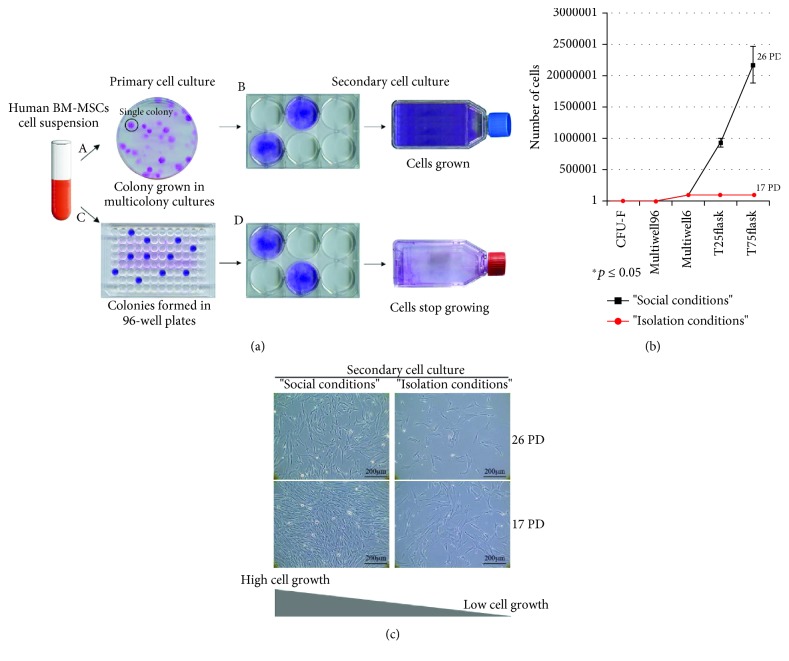
Clonal expansion depends on serum. (a) Samples of nucleated cells from primary BM-MSC cultures were plated and cultured either in 100 mm dishes at 1.6 cells/cm^2^ density or in 96-well plates at cloning dilution in FBS-supplemented culture medium. Colonies formed in 100 mm dishes (“social” conditions; A, B) could be further expanded. In contrast, colonies grown in 96-well plates at cloning dilution (“isolation” conditions; C, D) could not be further expanded. (b) Proliferation curve of BM-derived clonal cell strain grown in social context (black diagram) or in “isolation” conditions (red diagram). Five individual colonies formed in 96-well plates and 5 from multicolony cultures were tested for PD. (c) Colonies grown in 96-well plates underwent a lower number of PD, 16 (T75 flask), after which the cells became senescent forming colonies identifiable for their unusual, large and irregular shape compared to colonies grown in social context which could be further expanded to 22 PD (T75 flask). Images shown are representative results of secondary cell cultures expanded from colonies formed in social context or grown at cloning dilution. Results are expressed as the mean ± SD (*n* = 5). ^∗^*p* ≤ 0.05. Scale bars represent 200 *μ*m as indicated.

To investigate expansion abilities of BM MSC-initiated clones, we prepared secondary cultures from primary BM-MSC cultures. We plated 100 secondary BM-MSCs at 1.6 cells/cm^2^ in a 100 mm Petri dish or at a limiting density in 96-well plates in standard conditions. Samples from the same parent culture formed the same number of colonies under “social” [Figure 4(a), A] and “isolation” conditions [Figure 4(a), C]. However, when we monitored the ability of individual clones generated either in “isolation” or under “social” conditions to grow and expand, only the colonies exposed to “social conditions” during the initial phase of clonal expansion were able to preserve a potential for further growth [Figure 4(a), B and D]. Continuous passaging of individual colonies revealed dramatic differences of expansion capabilities between clones initially seeded under “social” or “isolation” conditions [Figure 4(a), A–D]. In particular, colonies formed under “social conditions” could be further expanded [up to 22-24 PD; Figure 4(b)], while colonies formed in 96 wells underwent senescence after few duplications [up to 22-26 PD; Figure 4(b)] and [14-17 PD; Figures 4(b) and 4(c)].

### 3.5. Promiscuity Rescues Proliferation of Growth-Impaired MSC Clones

**Figure 5:**
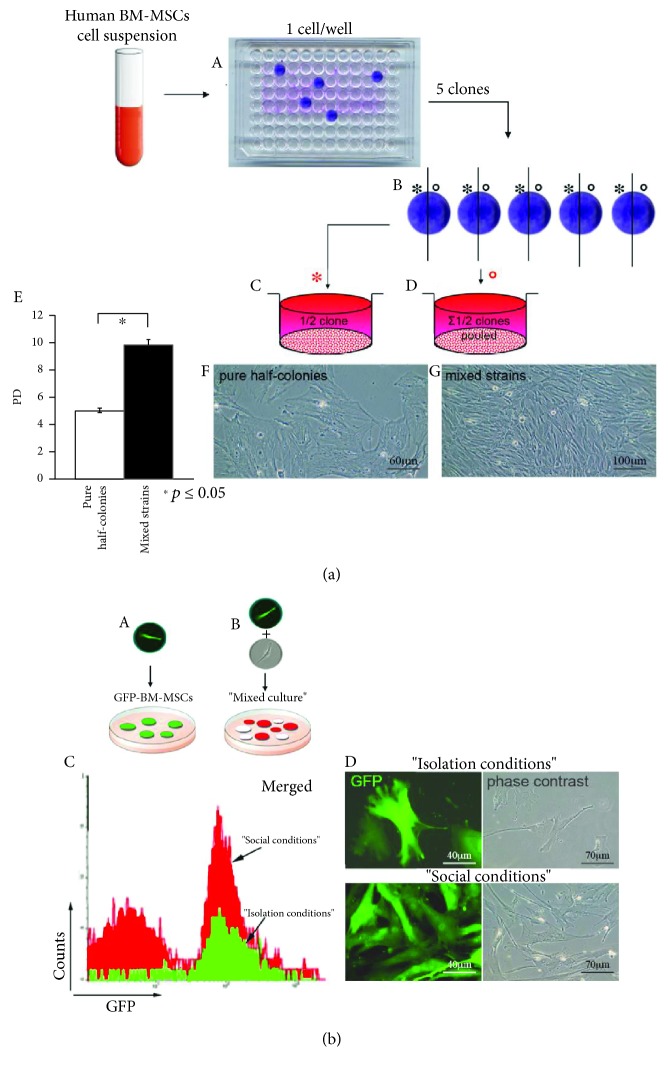
Clonal expansion is a biological property of MSC acquired in a social context. (a) Five individual colonies (6 × 10^3^ cells/colony) formed at cloning dilution in 96-well plates in FBS-supplemented culture medium (A) were trypsinized and split in half (B), and each half was replated either alone (C) or pooled with all other half clones (D). Individual pools were subdivided into 5 samples, each containing the same number of cells and replated in 6-well plates. Pure half colonies underwent senescence after 5 PD, whereas expansion of mixed strains, including the same number of cells as pure half colonies, continued for >10 PD (E). Culture images (F, G) shown are representative results. These experiments showed that promiscuous strains resumed the ability for efficient expansion, whereas pure clonal strains remained growth impaired (*n* = 5). (b) Assay for secondary CFE and growth potential of BM-MSC strains. 100 BM-MSCs transduced with GFP-lentiviral vectors were plated at clonal density either alone or together with 100 nontransduced BM-MSCs (A, B). After 14 days in culture, CFE was identical, but a 3-fold higher cell number was determined by FACS analysis (C), and a 2-fold higher PD was observed for GFP-BM-MSCs in the mixed cultures compared to the “pure” GFP-BM-MSCs (*n* = 5). Scale bars represent 40 *μ*m, 60 *μ*m, 70 *μ*m, and 100 *μ*m as indicated.

To further investigate the roles of initial social deprivation in limiting subsequent clonal expansion of individual clonogenic cells, we assessed whether expansion capabilities of clones initially grown in isolation could be rescued by exposing them to a promiscuous environment. To address this question, 5 clones were grown in isolation [Figure 5(a), A] in 96-well plates to subconfluency, trypsinized, and split in two halves [Figure 5(a), B]. From each clone, one half was replated in 6-well plates as a single clone [Figure 5(a), C]; the other half was pooled with all other half clones [Figure 5(a), D]. The pool was subdivided into 5 samples, each containing the same number of cells as each pure clonal strain and replated in 6-well plates. In this way, cells from growth impaired clones could be either exposed or not, to the influence of cellular promiscuity. Pure half colonies underwent senescence after 5 PD, whereas the expansion of mixed strains, including the same number of cells as pure half colonies, continued for >10 PD [Figure 5(a), E–G].

Next, 100 cell samples of primary BM-derived clone cultures stably transduced with GFP-lentiviral vectors were plated in 100 mm dishes (1.6 cells/cm^2^), either alone or together with 100 untagged GFP BM-MSCs [“mixed culture”; Figure 5(b), A and B]. Identical numbers of GFP^+^ colonies formed in either type of cultures were generated 14 days later. Although no differences were observed examining the number of GFP^+^ colonies in the two types of culture, the total number of GFP^+^ cells generated in mixed cultures was more than 2-fold higher as compared to that in BM-MSC cultures plated alone at clonal density; this indicates that the magnitude of initial clonal growth of individual clonogenic cells is influenced by the total number of cells initially present in culture [Figure 5(b), C and D]. These experiments showed that promiscuous strains resumed the ability for efficient expansion, whereas pure clonal strains remained growth impaired.

## 4. Discussion

The term “quorum sensing” is derived from Latin *quorum*; in politics, this is the number of votes that must be cast for an election or referendum to be valid. In biology, QS is defined as a process by which cells detect the accumulation of released signals and then change their behavior when signal concentration exceeds a threshold level. Cells communicate using chemical signaling molecules. Information provided by these molecules is critical for synchronizing activities of large groups of cells [1]. This chemical communication allows cells to monitor their environment and change behavior accordingly. This network of cell-cell cross-communication is integrated by cells, processed, and transduced to control gene expression. Identification of these chemical signals, receptors, target genes, and mechanisms of signal transduction involved in the QS network may help to shed light on cell-cell communication. This can be seen as a chemical vocabulary, and signaling networks are complex and often consist of multiple circuits organized in a variety of configurations, still to be identified. All these factors modulate mammalian cell behavior promoting development and differentiation and should be considered in future applications [16, 17]. QS is a system of stimuli/responses correlated to population density. However, QS is also activated by a single cell when seeded in an extremely enclosed space. Indeed, QS-regulated processes are relevant for both large cell colonies and single cells in confined spaces. Mechanisms of stem cell self-renewal are crucial to stem cell biology and regenerative medicine.

Historically, the very first evidence indicating the existence of stem cell postnatal progenitors for connective tissues originated from the seminal work of Friedenstein and coworkers, who were able to demonstrate that inherent osteogenic, adipogenic, and chondrogenic potential of unfractionated BM, known from earlier studies, could be ascribed to (a) a nonhematopoietic, stromal fraction and (b) single cells, capable of initiating clonal growth in a density-insensitive fashion [30–32]. The studies proposed here are not aimed at investigating niche-dependent and population-dependent mechanisms of MSC regulation but only at developing a series of hypotheses based on our observations. With these premises, we asked if there is any QS rule in clonogenicity and secondary expansion of BM-derived stromal MSCs.

Using a combination of time-lapse microscopy, permutation of culture conditions, and labeling methods, we observed that distinct seeding setup could affect biological properties including clonogenicity and secondary expansion of BM-derived mesenchymal stromal cells. Indeed, we observed that initiation of clonal growth and subsequent *in vitro* expansion of BM-MSC clonal stromal cell strains are biologically distinct events. Two developmental stages were recognized during the growth of each colony. In the first phase, lasting for the first week after plating, cells proliferate and migrate to define the area of the substrate to be covered by the monolayer (definition of territory). In the second phase, cells proliferate inside the previously marked territory, to generate a typical colony (filling up phase). However, clonogenicity of this subset of stromal adherent cells is strictly dependent on serum and independent of the social context. In contrast, clonogenicity is not supported by a serum-free medium. Further expansion of individual clones is affected by events emanating from a social context during initial growth. The ability of socially generated clones to develop an inherent potential for further growth suggests that effects of the *quorum* rule may operate in BM-MSC cultures and determine potential growth of clonal strains derived from single BM-CFU-F-MSCs. Acceptance of this paradigm identified for stromal BM-MSC biology might in general be useful to regulate BM-MSC expansion for clinical applications of skeletal regenerative therapies.

## 5. Conclusions

Our observations led us to hypothesize that some biological properties of clonal MSCs such as generation of discrete colonies and their expansion to larger single colony-derived population are differentially regulated. In the presence of serum, single clonal MSCs are able to generate discrete colonies independently of plating conditions. However, the capability to generate a larger pure clonal cell population seems to depend on reciprocal influences among different stromal cells. The influence of the social context is effective at the early stages of colony development, during which clonal cells commit to a potential larger expansion over time. Therefore, in analogy with bacterial biofilm, MSC populations can be considered “multicellular communities” providing single cells with distinct biological properties. The aim of this study was to evaluate the *ex vivo* amplification potential of BM-derived MSCs using defined culture conditions as growth stimulus. These data will be beneficial to set standards for tissue collection and MSC clinical-scale expansion, both for cell banking and for cell-based therapy settings. Studies on the QS cell-to-cell communication network system of *ex vivo* BM stromal MSC biological properties from functional commitment to growth abilities should be further explored, in perspective of refined clinical use for new therapeutic strategies.

## Figures and Tables

**Table 1:** Antibodies used for flow cytometry.

Antigen	Type	Label	Clone	Distributor
CD34 (gp 105-120)	MC	FITC	581	BD Biosciences
CD45 (LCA, T200)	MC	FITC	HI30	BD Biosciences
CD73	MC	PE	AD2	BD Biosciences
CD90 (Thy-1)	MC	APC	5E10	BD Biosciences
CD105 (Endoglin)	MC	PE	266	BD Biosciences
HLA-DR	MC	PE	L243	BD Biosciences
HLA-ABC	MC	FITC	G462.6	BD Biosciences

MC: mouse monoclonal; FITC: fluorescein isothiocyanate; PE: phycoerythrin; APC: allophycocyanin.

## Data Availability

All relevant data used to support the findings of this study are included within the paper.
